# Yellow fever mortality in Brazil: an age-period-cohort study

**DOI:** 10.11606/s1518-8787.2025059006721

**Published:** 2025-12-01

**Authors:** Lucas Casagrande Passoni Lopes

**Affiliations:** I Universidade de São Paulo. Faculdade de Medicina de Bauru. Bauru, SP, Brasil

**Keywords:** Yellow Fever, Mortality, Brazil

## Abstract

**OBJECTIVE:**

To analyze yellow fever mortality trends in Brazil, focusing on sexes differences and using an age-period-cohort model.

**METHODS:**

This ecological study analyzed yellow fever mortality data in Brazil from 1980 to 2019 sourced from Datasus. Population estimates were retrieved from the *Instituto Brasileiro de Geografia e Estatística* (Brazilian Institute of Geography and Statistics). Mortality data, including age, year of death, and cause (ICD-9: 060; ICD-10: A95), were analyzed using an age period cohort model. A Poisson distribution was assumed for mortality counts, and analyses were conducted using Holford’s method and its adaptations on R.

**RESULTS:**

The results show that the incidence rate peaked at younger ages, such as 30 years (0.010/100,000 individuals, 95%CI: 0.008/100,000 to 0.013/100,000), followed by a gradual declining trend with increasing age, reaching 0.007/100,000 individuals (95%CI: 0.005/100,000 to 0.010/100,000) at 50 years onward. Regarding period, a substantial increase in the adjusted hazard ratio occurred over time, especially in 2015 (13.923 [95%CI: 11.095 to 17.471]), suggesting a significant elevation when compared with previous periods. Cohort analysis showed a trend of increasing risk until 1960 (RR = 1.000), followed by a marked reduction for more recent cohorts, such as 2010: RR = 0.056 (95%CI: 0.028 to 0.112). Vaccination analysis showed alternating periods of significant increases and decreases in vaccination rates.

**CONCLUSIONS:**

Younger individuals showed higher mortality rates, with a gradual decline with advancing age. Period effects highlighted a pronounced resurgence in recent years, particularly during the 2015 epidemic, underscoring the influence of temporal factors such as outbreaks and vaccination campaigns. Cohort analysis showed a progressive decline in mortality risk among more recent birth cohorts, likely reflecting the impact of expanded immunization programs and improved public health measures. The proposed yellow fever vaccination trends in Brazil may explain some of the observed patterns.

## INTRODUCTION

Yellow fever (YF) is an acute viral hemorrhagic disease caused by the YF virus^1^. This zoonotic disease primarily affects humans and non-human primates and is transmitted via the bite of infected mosquitoes such as *Haemagogus*, *Sabethes*, and *Aedes aegypti* ^1^.

YF often shows a biphasic clinical course^1^. Its initial acute phase includes nonspecific symptoms such as fever, chills, and headache^1^. In about 15% of cases, the disease progresses to a severe, life-threatening phase showing high fever, jaundice, bleeding, shock, and multi-organ dysfunction^1^. This toxic phase is associated with a high case fatality rate, ranging from 20% to 60%, particularly among those with hemorrhagic symptoms and liver failure^1^.

YF is endemic to tropical regions of Africa and South America, with periodic outbreaks posing significant health, economic, and social burdens^2^. Despite global vaccination efforts, the World Health Organization estimates that approximately 200,000 cases and 30,000 deaths occur annually worldwide^2^. The costs associated with YF outbreaks go beyond healthcare expenses, encompassing economic losses due to workforce morbidity, mortality, and the implementation of large-scale emergency vaccination campaigns^2^.

The epidemiology of YF in Brazil reflects a complex interplay between environmental, socioeconomic, and public health dynamics^3^. Documented since the Brazilian colonial period, YF emerged as a significant public health threat, particularly in urban centers, where mosquitoes facilitated transmission^3^. Efforts to combat the disease grew in the early 20th century following landmark discoveries linking mosquito vectors to disease propagation^3^. The advent of the 17D vaccine in the 1930s revolutionized its prevention, with mass immunization campaigns drastically reducing outbreaks in endemic regions^3^. However, sporadic epizootics and human cases, as the ones from 2016 to 2019, persist in sylvatic cycles, underscoring the need for sustained vaccination coverage and surveillance^3^. Recent efforts have focused on expanding vaccine access to at-risk populations and mitigating urban transmission risks by vector control initiatives and public health education^3^.

The dynamic epidemiological profile of the disease and its periodic outbreaks and shifts in transmission zones, and the emergence of new viral lineages justifies an age period cohort (APC) study on YF mortality. This study design can comprehensively analyze temporal trends and find at-risk groups based on age, historical periods of increased transmission, and birth cohort characteristics. Therefore, this study aims to evaluated YF mortality in Brazil by an APC model.

## METHODS

### Study Characterization

This is an ecologic study that evaluated YF mortality in Brazil by an APC model.

### Data Source

All data were extracted from *Departamento de Informação e Informática do Sistema Único de Saúde* (Datasus).

The data were categorized into demographic and clinical groups. The demographic data refer to the total population in the evaluated Brazilian regions in the observed period. Such data were obtained from the census and demographic projections of the *Instituto Brasileiro de Geografia e Estatística* (IBGE - Brazilian Institute of Geography and Statistics), which is available on the platform above.

Patients’ clinical data refer to the age at which they were deceased, the year of their death, and the cause of their death. Patients whose cause of death was identified by the code 060 according to the standardization of the International Classification of Diseases in its ninth edition (ICD9) and codes A95 according to ICD10 were chosen for this study.

Datasus is synchronized with the Brazilian Mortality Information System, an online, public, and open-access platform that gather the data on death certificates in Brazil. The system provides available data for epidemiological evaluation of various diseases, including YF, stratifying them by year, macroregion, and other variables.

A 40-year period (1980–2019) was evaluated since it was the one in Datasus with all the data of interest.

Data on YF vaccination in Brazil from 1994 to 2022 were derived from national immunization databases, including *Programa Nacional de Imunizações*. These vaccines, predominantly live-attenuated virus types, are recommended for individuals residing in or traveling to endemic areas. A single dose confers lifelong immunity. Vaccination is primarily indicated for individuals aged nine months and older, with specific guidelines for adults and older adults depending on risk-benefit assessments. The dataset on vaccine distribution provides valuable insight into coverage trends and demographic patterns of immunization, which are crucial for understanding disease prevention efforts.

### Data Analysis

The data were arranged in Excel^®^ spreadsheets according to the Lexis diagram.

Birth cohorts were calculated by subtracting the year of hospitalization from patients’ age at death according to the classical method. The periods were categorized as 1980–1984, 1985–1989, 1990–1994, 1995–1999, 2000–2004, 2005–2009, 2010–2014, and 2015–2019. Age was categorized in five-year groups as 0–14, 15–19, 20–24, 25–29, 30–34, 35–39, 40–44, 45–49, 50–54, 55–59, 60–64, 65–69, 70–74, and 75 years or older.

Assuming that the number of deaths resulting from a counting process follows a Poisson distribution, APC can assign a more satisfactory distribution for the response variable and consider several forms for the often non-linear relationship between the number of deaths and the explanatory variables (age, period, and cohort). Nonetheless, the model can cause the overdispersion of mortality rates, in which case another distribution and other data analysis method should be used.

The effects were analyzed using the APC model proposed by Holford^4^ and adapted by Clayton and Schifflers^5^, and Carstensen^6^. The mortality rate (𝜆*ijk*) for age (*i*), period (*j*), and cohort (*k*) was modeled according to the formula below. The μ represents the global average mortality rate; 𝛼, the average age effect; 𝛽, the average period effect; and 𝛾, the average cohort effect.


log⁡(λijk)=μ+αi+βj+γk

The relative risks (RR) in this study were derived from the APC model, which considers the independent effects of age, temporal periods, and birth cohorts on YF mortality. The adjusted rates represent the average rates for the analyzed periods rather than a specific timeframe, more comprehensively evaluating long-term trends. This methodological approach can disentangle the complex interplay between demographic, temporal, and generational factors, providing insights into mortality patterns across diverse subpopulations and timeframes. By integrating this model, this study ensures that observed risks are adjusted for underlying population dynamics and temporal changes, enhancing the robustness and interpretability of its results.

Likelihood ratio tests were used to compare submodels to assess the effects of age, period, and cohort on mortality rates. Such submodels were adjusted in a conveniently organized sequence to provide the tests for the mentioned effects as a comparison between them. Based on these comparisons, made by using the Akaike information criterion (AIC), the best model was obtained. The adequacy of the final model fit was verified via deviance statistics. The analysis was performed on R^®^, 4.4.0.alpha, by the function Epy.

Joinpoint regression, a statistical method to identify changes in trends within a time series, was applied to evaluate shifts in vaccination coverage and their potential relationship with mortality trends in the APC analysis of YF mortality in Brazil. This method detects inflection points—moments in which a significant change in the trend occurs—and estimates annual percentage changes (AC) for each segment. By aligning vaccination trends with the findings of the APC model, we observed that periods of increased vaccine coverage often coincide with declines in cohort-specific RR of mortality, particularly for individuals who were born after the introduction of widespread vaccination campaigns. Although the vaccine data period (1994–2022) fails to encompass the entire scope of the APC study, it provides critical context for interpreting mortality trends. This suggests that enhanced vaccine coverage may have played a pivotal role in mitigating age-specific and cohort-based risks over recent decades. This integrative approach underscores the interplay between public health interventions and epidemiological patterns, highlighting the enduring impact of vaccination on YF control.

### Ethics

This study required no ethical probation for its development.

## RESULTS

This study tested several data analysis and correlation methodologies, including linear and quadratic regressions, which showed 0.632 and 0.317 model adequacy p-values, respectively. This study chose the analysis with Splines as its model due to its statistical significance (p < 0.01). Starting from the simplest age-only model, with a 1,321.33 AIC, the inclusion of drift effects (age-drift model) significantly improved fit, reducing the AIC to 890.07 (ΔDev = 433.27; p < 0.01). Further refinements incorporating cohort effects (age-cohort model) yielded a 794.40 AIC, with an additional improvement in deviance (ΔDev = 101.66, p < 0.01). The APC model ultimately showed the best fit, achieving the lowest AIC (565.10) and a 231.30 deviance reduction (p < 0.01) when compared to its nested counterparts, underscoring the significant contributions of period and cohort effects. The likelihood ratio tests consistently supported the hierarchical progression, confirming that each additional term contributed meaningfully to the fit of the model. This study conducted residual analyses and deviance diagnostics to evaluate the adequacy of the final APC model. Its deviance totaled 229.13 with 102 degrees of freedom, indicating a satisfactory fit to the observed data. This research inspected the distribution of residuals to ensure that no systematic patterns remained unexplained, further supporting the validity of the model. These diagnostic steps underscored the capacity of the APC model to effectively capture the interplay of age, period, and cohort effects on YF mortality, providing a robust framework for interpreting temporal trends and demographic variations.

**Figure 1:**
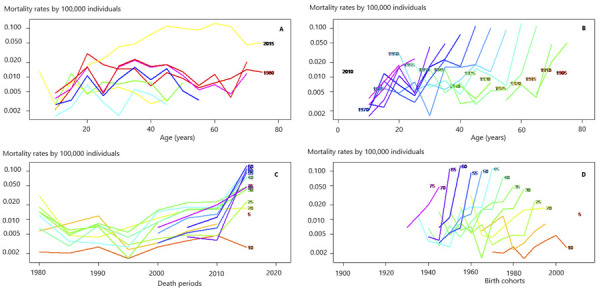
Mortality trends by yellow fever disease in Brazil from 1980 to 2019 regarding age, period, and cohort.

**Figure 2:**
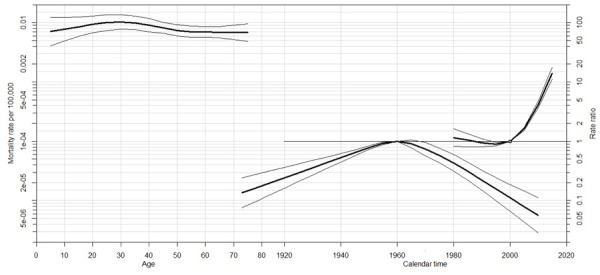
Summary of the main results observed in an age, period, and cohort model of mortality trends by yellow fever in Brazil from 1980 to 2019.

Source: *Departamento de Informação e Informática do Sistema Único de Saúde* (Datasus). Note: the figure shows, in the upper and left corner, the mortality rates by 100,000 individuals, expressed in the left y-axis for each age group represented in the x-axis. The right lower corner shows the rate ratio in the right y-axis (which expresses a specie of relative risk for birth cohorts) and the x-axis represents periods. The biggest parable represents the births cohorts and the smallest, the period.

In the first age groups, the mortality rate increases with age up to around 30 years, peaking at 0.010/100,000 individuals (95%CI: 0.008/100,000 to 0.013/100,000), preceding a slight decrease to 0.007/100,000 individuals (95%CI: 0.005/100,000 to 0.010/100,000) in the age group close to 50 years. This rate persists in the groups described in Table 1. The period relative risk showed a significant increase in recent periods. In 2015–2019, the period relative risk reached 13.923 (95%CI: 11.095 to 17.471), indicating a significant increase in mortality when compared with the base year (2000) (Table 2). The cohort of 1960 serves as the reference point (RR = 1.000). For cohorts preceding 1960, an incremental rise in RR occurred—reflecting a progressively heightened risk—from 0.136 (95%CI: 0.076 to 0.243) in 1905 to a 0.927 peak (95%CI: 0.881 to 0.975) in 1955. Conversely, cohorts born after 1960 showed a strikingly consistent reduction in RR, in which the risk diminished substantially in more recent generations. For instance, the RR decreased to 0.431 (95%CI: 0.312 to 0.595) for the 1980 cohort and continued to decline dramatically, reaching 0.056 (95%CI: 0.028 to 0.112) by the 2010 cohort (Figures 1 and 2 and Table 3).

**Table 1:** Yellow fever mortality rate by 100,000 individuals, stratified by age groups.

Age groups in years	Mortality rates by 100,000 individuals	95% confidence interval
0–14	0.007	(0.004 to 0.012)
15–19	0.008	(0.006 to 0.012)
20–24	0.009	(0.007 to 0.013)
25–29	0.009	(0.007 to 0.013)
30–34	0.010	(0.008 to 0.013)
35–39	0.010	(0.008 to 0.013)
40–44	0.009	(0.007 to 0.012)
45–49	0.008	(0.007 to 0.010)
50–54	0.007	(0.006 to 0.009)
55–59	0.007	(0.006 to 0.009)
60–64	0.007	(0.006 to 0.009)
65–69	0.007	(0.006 to 0.009)
70–74	0.007	(0.005 to 0.009)
≥ 75	0.007	(0.005 to 0.010)

Source: *Departamento de Informação e Informática do Sistema Único de Saúde* (Datasus).

**Table 2:** Yellow fever mortality relative risk stratified by periods groups.

Period	Mortality relative risk	95% confidence interval
1980–1984	1.145	(0.814 to 1.610)
1985–1989	1.042	(0.812 to 1.338)
1990–1994	0.949	(0.810 to 1.112)
1995–1999	0.890	(0.829 to 0.956)
2000–2004	1	-
2005–2009	1.611	(1.527 to 1.701)
2010–2014	4.137	(3.652 to 4.687)
2015–2019	13.923	(11.095 to 17.471)

Source: *Departamento de Informação e Informática do Sistema Único de Saúde* (Datasus).

**Table 3:** Yellow fever mortality relative risk stratified by birth cohort groups.

Birth’s year (begin of birth cohort)	Mortality relative risk	95% confidence interval
1905	0.136	(0.076 to 0.243)
1910	0.165	(0.098 to 0.277)
1915	0.200	(0.126 to 0.317)
1920	0.243	(0.163 to 0.363)
1925	0.295	(0.210 to 0.415)
1930	0.358	(0.270 to 0.474)
1935	0.434	(0.347 to 0.543)
1940	0.527	(0.447 to 0.622)
1945	0.640	(0.573 to 0.715)
1950	0.777	(0.727 to 0.830)
1955	0.927	(0.881 to 0.975)
1960	1	-
1965	0.910	(0.785 to 1.055)
1970	0.740	(0.572 to 0.956)
1975	0.577	(0.437 to 0.762)
1980	0.431	(0.312 to 0.595)
1985	0.312	(0.220 to 0.443)
1990	0.222	(0.151 to 0.327)
1995	0.158	(0.101 to 0.246)
2000	0.112	(0.067 to 0.187)
2005	0.079	(0.044 to 0.144)
2010	0.056	(0.028 to 0.112)

Source: *Departamento de Informação e Informática do Sistema Único de Saúde* (Datasus).

YF: yelow fever. Source: *Departamento de Informação e Informática do Sistema Único de Saúde* (Datasus).Note: in Figure 1, graph A, in the upper left corner shows, on its y-axis (vertical), the mortality rate due to YF for every 100,000 individuals. The X-axis (horizontal) specifies the age at which the individuals died. Each line on the graph represents a specific period. Graph B in the upper right corner shows, on its Y-axis (vertical), the mortality rate due to YF for every 100,000 individuals. The X-axis (horizontal) shows the age at which individuals. Each line on the graph represents a specific birth cohort. The graph C in the lower left corner shows, on its Y-axis (vertical), the mortality rate due to YF for every 100,000 individuals, whereas on the X-axis (horizontal), the evaluated periods. Each line on the graph represents a specific age group. Finally, graph D in the lower right corner shows, on its Y-axis (vertical), the mortality rate due to YF for every 100,000 individuals, whereas on the X-axis (horizontal), the birth cohorts. Each line on the graph represents a specific age group.

**Figure 3:**
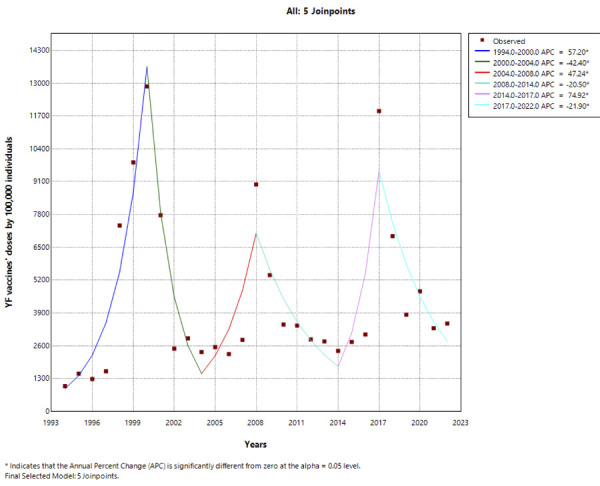
Temporal trends in yellow fever vaccination in Brazil from 1994 to 2022.

APC: annual percentage change; YF: yellow fever. Source: *Departamento de Informação e Informática do Sistema Único de Saúde* (Datasus). Note: The figure represents the samples of YF vaccines that were applied for each 100,000 Brazilian individuals in the y-axis for each year in the x-axis. Each square represents a graph point and the lines indicate the observed trends throughout the period.

The joinpoint regression analysis of YF vaccine coverage in Brazil from 1994 to 2022 showed significant fluctuations in AC across distinct time periods. From 1994 to 2000, a sharp increase in vaccination rates occurred, with an AC of 57.20 (95%CI: 37.82 to 101.21; p < 0.001). This preceded a marked decline from 2000 to 2004, with an AC of −42.40 (95%CI: -59.73 to -30.08; p = 0.002). Subsequently, the period from 2004 to 2008 saw a resurgence in vaccination rates, with an AC of 47.24 (95%CI: 20.37 to 123.85; p = 0.003). However, a declining trend emerged from 2008 to 2014, with an AC of −20.50 (95%CI: −47.94 to −13.65; p = 0.003). Notably, from 2014 to 2017, vaccination rates sharply increased again, with an AC of 74.92 (95%CI: 28.91 to 123.03; p = 0.002), before another significant decline from 2017 to 2022, with an AC of −21.90 (95%CI: −37.13 to −14.26; p = 0.002). These trends underscore dynamic changes in vaccination efforts, potentially reflecting responses to outbreaks and public health campaigns during this period (Figure 3).

## DISCUSSION

### Age

Our APC analysis highlights a complex relationship between age and mortality rates in YF. Mortality initially rises with age, peaking around 30 years, before slightly declining and stabilizing in older groups. This trend contrasts with previous studies showing higher mortality in older individuals, often attributed to immunosenescence and chronic inflammation (“inflammaging”)^7^. Age-related immune dysfunction (including reduced T-cell activity, impaired antibody production, and elevated cytokines such as IL-6 and TNF-α) is associated with severe outcomes, such as cytokine storms and multi-organ failure^7,8^. Additionally, comorbidities prevalent in older adults, such as diabetes and liver disease, exacerbate the impact of YF^8,9^.

Our findings may reflect the influence of vaccination efforts, as per our joinpoint regression analysis of YF vaccine coverage. Periods of increased vaccine uptake, such as from 1994–2000 and 2014–2017, likely contributed to stabilizing mortality rates in middle-aged and older groups^10^. However, declines in coverage during 2000–2004 and 2017–2022 suggest vulnerabilities that may have sustained mortality risks. Although the live-attenuated YF vaccine elicits robust immune responses, older adults often show reduced immunogenicity, emphasizing the need for age-targeted vaccination strategies and booster doses^11^.

Hormonal shifts with age, such as decreased estrogen and testosterone, further influence immune responses to YF virus and vaccine efficacy^12,13^. Estrogen enhances immune activity, whereas testosterone can suppress it, with their decline potentially weakening the immune response in older adults^12,13^. These factors, combined with declining vaccine coverage in certain periods, may explain the observed mortality patterns.

### Period

The period-relative risk analysis in this study showed a significant increase in YF mortality in recent years, particularly from 2015 to 2019, when the period relative risk reached 13.923 (95%CI: 11.095 to 17.471), reflecting a stark rise when compared with the base year (2000). This trend aligns with the resurgence of YF epidemics in Brazil driven by socio-environmental shifts such as deforestation, urban expansion, and closer human interactions with sylvatic transmission cycles^3,8,11^. These changes increased exposure to infected non-human primates and sylvatic mosquito vectors, especially in previously unaffected regions^3,8,11^. Consequently, the expansion of YF to areas with historically lower vaccination coverage likely contributed to elevated mortality rates as populations in these regions were inadequately protected against the virus^3,8,11^.

Fluctuations in vaccination efforts during this period provide critical context for these mortality trends. Our joinpoint regression analysis highlights periods of sharp increases and declines in vaccination coverage from 1994 to 2022. For instance, significant increases in coverage occurred from 1994 to 2000 and again from 2014 to 2017. However, declines from 2000 to 2004 and from 2017 to 2022 produced periods of vulnerability, particularly as outbreaks worsened^5,6,14^. These fluctuations suggest that while vaccination campaigns were effective during outbreaks, sustaining high coverage proved challenging, especially in remote or newly affected areas^5,6,14^.

The resurgence of YF from 2016 to 2019 exemplifies the interplay between vaccine coverage and mortality risk^9^. Despite efforts to deploy mass vaccination campaigns during this time, logistical challenges, vaccine shortages, and delayed responses hindered the ability of the program to achieve sufficient coverage in high-risk populations^9^. Urbanized regions, including São Paulo and Minas Gerais, in which large outbreaks occurred, faced heightened risks due to their dense populations and the mobility of sylvatic vectors^9^. These conditions exacerbated the mortality burden as healthcare systems struggled to meet the demands of diagnosing and treating severe YF cases, further exposing vulnerabilities in public health infrastructure^9^.

The observed trends underscore the critical role of vaccination in mitigating YF mortality while highlighting the need for consistent and proactive immunization strategies^7,8^. Although individuals’ immune responses and vaccine efficacy may vary, maintaining high coverage remains essential, particularly in endemic and high-risk regions^7^. Moving forward, targeted approaches to vaccination, improved logistical planning, and enhanced surveillance systems are vital to address ecological and epidemiological challenges^8^. By bridging gaps in vaccination and preparedness, Brazil can better mitigate the impacts of future outbreaks and reduce YF-related mortality^7,8^.

### Cohort

The relationship between birth cohorts and YF mortality reflects a dynamic interplay of epidemiological shifts and vaccination policies over time in Brazil^12,13^. Cohorts prior the advent of widespread YF immunization were shaped by the historical context of limited public health interventions, leaving them more vulnerable during outbreaks^12,13^. Specifically, individuals born before 1940, when urban YF transmission was eradicated in Brazil, likely missed early-life vaccination opportunities as the country shifted its focus away from the disease^12^. This gap in immunity left these cohorts at heightened risk for severe outcomes and mortality during later-life exposure to the virus, particularly if they migrated to or visited newly endemic areas^12^. Such generational disparities underscore the critical influence of temporal vaccination policies on susceptibility and mortality risk within different cohorts^12,13^.

In contrast, cohorts born after the 1980s benefitted significantly from enhanced vaccination strategies^9,10^. The gradual integration of YF immunization into the Brazilian national vaccination schedule—particularly following the severe outbreaks of the 1990s—marked a pivotal shift in proactive public health measures^9^. By prioritizing vaccination in endemic regions and at-risk populations, younger cohorts experienced substantial reductions in YF mortality risk^9^. These improvements are reflected in the declining RR observed in post-1980 cohorts, with a dramatic reduction in mortality risk in the 2010 cohort^10^. This generational advantage highlights the protective effect of the timely and widespread vaccination policies that effectively reduced susceptibility to severe disease across the population^10^.

However, the 2016–2019 outbreaks showed vulnerabilities that persist within certain cohorts, especially those born in regions not previously designated as at-risk for YF^11^. The geographical expansion of the virus into urbanized southeastern Brazil exposed populations that had been excluded from routine immunization efforts^11^. For these cohorts, particularly those born during periods of reactive rather than routine vaccination strategies, gaps in immunity became starkly evident^11^. This underscores the importance of re-evaluating historical vaccination coverage and addressing geographical disparities in future public health planning^11^.

Another critical consideration refers to the phenomenon of waning immunity within older cohorts. While the YF vaccine confers long-term protection, evidence suggests that immunity may fade over decades, especially in individuals vaccinated early in life^7,8^. This raises concerns for cohorts vaccinated in childhood during earlier mass campaigns as they may face increased susceptibility to severe disease in later life^7,8^. The interplay between natural aging, immunity fade, and cohort-specific vaccination timelines suggests a need for targeted booster policies, particularly for older adults who remain at risk despite prior immunization^7,8^.

The dynamic trends in vaccination coverage further illuminate the complex relationship between vaccination efforts and YF mortality^6,14^. The joinpoint regression analysis of YF vaccine coverage from 1994 to 2022 showed periods of rapid expansion and significant decline in vaccination rates^6^. Periods of sharp increases, such as from 1994 to 2000 or from 2014 to 2017, likely reflect responses to outbreaks or public health campaigns aimed at curbing viral spread^6^. Conversely, periods of decline, such as those from 2000 to 2004 and 2017 to 2022, may indicate waning public awareness, resource constraints, or competing health priorities^14^. These fluctuations highlight the challenges of maintaining consistent vaccine coverage and emphasize the potential risks of reduced vaccination momentum in perpetuating cohort-specific vulnerabilities^14^.

Together, these findings underscore the necessity of a multifaceted approach to YF control, incorporating routine immunization, booster campaigns for older cohorts, and geographically targeted interventions^7^. By addressing the unique vulnerabilities of specific birth cohorts and ensuring sustained vaccination coverage, public health authorities can effectively reduce the risk of future outbreaks and mitigate mortality across generations^7^.

### Strengths and Limitations

One primary limitation of this study refers to its use of aggregated data, which may obscure individual-level variations and lead to ecological fallacies since group-level associations may not hold true for individuals. The reliance on secondary data from the Datasus database could also introduce biases due to inaccuracies or incompleteness in the recording of mortality or demographic information. Furthermore, the APC model, while sophisticated, faces inherent challenges such as the identifiability problem, in which the age, period, and cohort effects are confounded and difficult to disentangle completely. Thus, definitively interpreting the results may configure a challenge. Another limitation stems from this study ignoring the specificities of sex and Brazilian macroregions.

Despite these limitations, several factors justify the use of this methodology. The APC model is a well-established approach for understanding trends in mortality over time, offering a structured way to assess the interplay between age, period, and cohort effects. By using data spanning 40 years, this study benefits from a long observational window that strengthens its analysis of temporal trends. The choice of Datasus as a source is also justified given its comprehensive coverage and accessibility, providing a reliable foundation for evaluating large-scale epidemiological phenomena in Brazil. Additionally, by focusing on YF mortality, which has specific ICD codes, the study enhances data specificity and minimizes misclassification.

The strengths of this study lie in its robust statistical methodology and large population-based approach. The use of the APC model offers nuanced insights into how YF mortality has evolved over time across age groups and birth cohorts, uncovering important patterns that would otherwise remain hidden in simpler models. The chosen comprehensive dataset, spanning four decades, provides a significant amount of data, enhancing the reliability of our findings. Furthermore, this study found key trends in mortality risks across periods and cohorts for men and women, contributing to the understanding of YF mortality in Brazil and potentially informing public health strategies aimed at mitigating these risks.

## CONCLUSIONS

This study highlights the influence of age, period, and cohort variables on the analyzed incidence rates, evincing significant variations over time. The risk peaks at younger ages and in recent periods suggest the need for interventions focused on the most affected age groups and on the temporal evolution of risk. ‘Moreover, the decrease in the risk ratio in younger cohorts indicates that changes across generations, possibly related to environmental, behavioral, or public health policy factors, may contribute to this reduction. Furthermore, Brazil shows an inconstant pattern in YF vaccination, which may reflect its outbreaks and mortality. These findings reinforce the importance of dynamic public policies adapted to demographic and temporal characteristics to mitigate future risks and promote population health.

## Data Availability

Raw data can be acesses at: https://datasus.saude.gov.br Available from: https://datasus.saude.gov.br.
